# Misinformation mayhem: the effects of TikTok content on ADHD knowledge, stigma, and treatment-seeking intentions

**DOI:** 10.1007/s00787-025-02769-8

**Published:** 2025-06-05

**Authors:** Ashley Schiros, Nick Bowman, Kevin Antshel

**Affiliations:** Syracuse University, Syracuse, United States

**Keywords:** ADHD, Misinformation, Social media, Treatment-seeking, College-students

## Abstract

Attention Deficit/Hyperactivity Disorder (ADHD) content on TikTok is popular among college students, yet ADHD misinformation is prevalent on TikTok. This study aims to experimentally investigate the effects of TikTok ADHD misinformation content on ADHD knowledge, stigma, and treatment-seeking intentions. An experimental design assessed the impact of ADHD (mis)information among treatment-naïve college students. A pilot phase was completed to develop TikTok stimuli, using a systematic content analysis, and provide initial evidence of feasibility. In the main study, participants (*N =* 490) were randomly assigned to one of three conditions (accurate ADHD information, ADHD misinformation, control) to view TikTok content. A baseline measure of ADHD knowledge was completed pre-content-viewing. Following content-viewing, participants completed measures assessing ADHD-related knowledge, stigma, and treatment-seeking intentions. Participants exposed to ADHD misinformation exhibited significantly less accurate ADHD knowledge, but higher confidence in their ADHD knowledge post-content-viewing. Participants exposed to accurate ADHD content exhibited significantly more ADHD knowledge and confidence in their knowledge post-content-viewing. The ADHD misinformation group reported higher intentions to seek both evidence-based and non-evidence-based ADHD treatment. No significant effects of content condition on ADHD stigma were found. Perceived entertainment of TikTok content was significantly associated with ADHD knowledge and treatment-seeking intentions. TikTok ADHD misinformation decreases ADHD knowledge yet increases confidence in that knowledge as well as ADHD treatment-seeking intentions. These findings provide an essential first step in understanding the potential harms of TikTok misinformation on the individual and public levels.

**Supplementary Information:**

The online version contains supplementary material available at 10.1007/s00787-025-02769-8.

Attention Deficit/Hyperactivity Disorder (ADHD) online content is growing increasingly popular, especially on the social media platform TikTok. TikTok is a highly trafficked social media platform with 1.7 billion monthly users, the largest demographic of whom are college age (18–24 years old) [1]. There are over 28 billion views on *#ADHD* videos on TikTok, and social media is among the most frequently used sources to search for mental health information [2], and, specifically, ADHD-related information [3]. Despite the near ubiquity of TikTok use among college students and high volume of ADHD-content on the platform, TikTok frequently provides and promulgates misinformation, defined as *unintentionally* false information [4], about ADHD [5–7]. Two recent content analyses of ADHD-content on TikTok found that the majority highly viewed #*ADHD* videos on the platform contained misinformation [6, 8]. Thus, there is a high probability that college students encounter ADHD misinformation on TikTok, and it is important to understand the implications viewing this misinformation on ADHD-related knowledge, stigma, and treatment-seeking intentions [9, 10].

Theoretical models of treatment-seeking behavior predict that decreasing accurate ADHD knowledge will result in *reduced* treatment-seeking for ADHD [11]; however, paradoxically, recent global trends denote *increased* treatment-seeking for ADHD [12], including national shortages of stimulant medication in the United States and United Kingdom [13, 14] and all-time high waitlist lengths for ADHD assessment and psychotherapy [13, 14]. Given the discrepancy between theoretical predictions and the current surge in ADHD treatment-seeking, understanding how ADHD misinformation from TikTok might affect treatment-seeking intentions is crucial. Increased ADHD awareness from online content may influence individuals to seek diagnosis and treatment. Yet, no research to date has implemented an experimental design or used a theoretical model to consider how ADHD TikTok content impacts ADHD-related knowledge, ADHD stigma and treatment-seeking intentions.

## Theoretical framework

Extant research on ADHD treatment-seeking is largely atheoretical and focuses almost exclusively on parental decision-making about their child [15]. The Information-Motivation-Behavioral (IMB) Skills Model may help to understand how misinformation affects ADHD-related knowledge, stigma, and individual treatment-seeking intentions among college-age adolescents. The IMB model posits that engagement in health-promoting behaviors is determined by health-related information, motivation, and behavioral skills [11]. Health-related information includes health facts; according to IMB, accurate knowledge is a requirement to engage in health-promoting behavior [11]. ADHD-relevant health information includes accurate information regarding symptoms and treatments. ADHD misinformation most directly relates to this IMB construct. According to IMB, motivation (e.g., low ADHD stigma) is a critical determinant of engaging in health-promoting behavior [11]. Low stigma has previously been used as a proxy for motivation in IMB [16]. Behavioral skills, encompasses perceived or actual skills pertaining to health-promoting behaviors, including ability to identify and engage in evidence-based treatment. Behavioral skills involve seeking evidence-based ADHD and avoiding non-evidence-based treatment. Relating to TikTok, ADHD misinformation may reduce ADHD knowledge, thereby increasing stigma and decreasing behavioral skills and treatment-seeking. Although the IMB model predicts decreased treatment-seeking [11], increased ADHD treatment-seeking is occurring globally [12, 14]. Perceived TikTok content credibility may help to understand discrepancy between IMB predictions and the recent lack of accessibility to ADHD treatments.

### Perceived credibility

According to leading persuasion theories, messaging perceived as highly credible by the consumer is more likely to engender changes in consumer attitudes and behaviors [17]. Expertise, trustworthiness, similarity, and entertainment are key facets of perceived credibility related to behavior change [18]. TikTok content that may contain misinformation is often perceived as entertaining, and entertainment is a common motivator for sharing misinformation [19]. Therefore, the relationship between ADHD content on TikTok and IMB variables may be influenced by perceived credibility, especially entertainment. Applying both the IMB model and persuasion credibility variables to the context of ADHD TikTok misinformation may provide theory-driven explanations for the effects of ADHD misinformation on college students and help to explain the discrepancy between IMB predictions (less treatment seeking) and the recent surge in ADHD treatment seeking.

### College student mental health treatment-seeking

The college student population is particularly important to study regarding exposure to ADHD misinformation given their high TikTok use and reliance on social media for mental health information [2]. Previous ADHD treatment-seeking research highly focuses on parental treatment-seeking for their child [15]. Far less is known about factors contributing to college students seeking healthcare treatment for themselves, often for the first time in their lives, and his transition to independently making healthcare decisions is often complex [20]. Moreover, the density of ADHD misinformation on TikTok may impact treatment-making decisions among college students. Although social media provides an opportunity for the public to discuss mental health, misinformation is associated with increased stigma [21], engagement in non-evidence based treatments [22], and misallocation of healthcare resources [23]. Likewise, TikTok creates opportunities for spreading, albeit non-deliberately, misinformation, which can reduce engagement in health promotive behaviors and/or increase engagement in non-evidence based treatments [24, 25]. Specific to ADHD, poorer understanding of ADHD is associated with higher acceptance of non-evidence-based treatments and lower acceptance of evidence-based treatments (e.g., stimulant medication) for ADHD [26] and decreased reliance on healthcare providers’ recommendations [25].

In summary, there is currently an inundation of highly-viewed ADHD content and misinformation on TikTok. Misinformation perpetuates misconceptions surrounding mental health and contributes to misallocation of health resources. However, there is a lack of research examining (a) how ADHD information on TikTok impacts ADHD knowledge, stigma, and treatment-seeking intentions; and (b) if perceived content credibility impacts these ADHD-related outcomes. The present study is innovative in its implementation of an experimental design comparing the effects of exposure to TikTok ADHD misinformation and accurate ADHD information on ADHD knowledge, stigma, and treatment-seeking intentions.

The goals of this project were to: (1) determine the effects of misinformation and accurate ADHD TikTok content on ADHD knowledge, stigma of ADHD, and treatment-seeking intentions; (2) investigate the extent to which dimensions of content perceived credibility influence ADHD knowledge, ADHD stigma, and ADHD treatment-seeking intentions.

## Methods

The current study utilized an experimental design with three conditions (ADHD misinformation, accurate ADHD information, control). Prior to completion of the main study, a pilot phase was conducted to develop stimuli and provide initial evidence of feasibility. The pilot study methods and results are presented in Supplementary File 1. Study procedures were approved by Syracuse University's Institutional Review Board (IRB#23–311). All study materials and stimuli are shared freely through an Open Science Foundation project space: https://osf.io/v5kwr/?view_only=c3090e2e6fd14378ba7459c9380a51dd.

### Study participants

A total of *N =* 490 college student participants were recruited from Syracuse University. Recruited participants met inclusion/exclusion criteria for this study. Inclusion criteria were: (1) aged 18–25, (2) English-fluent, (3) social media user, (4) no history of formal ADHD diagnosis and/or treatment, and (5) endorsing 2 + current ADHD symptoms occurring ‘often’ or ‘very often’. The study targets treatment-naïve emerging adults who report current ADHD symptoms.

The mean age of participants was 19.09 (SD = 1.30). Most participants were in their first year of college (56.7%) and identified as White (59.6%) and female (69.6%). 90.9% of participants reported having and actively using a TikTok account, and the mean amount of time reported spent on TikTok was one to two hours per day. The level of ADHD symptoms (*M =* 47.79, SD = 11.61) in this sample was consistent with the report of adults aged 18–29 diagnosed with ADHD and well above the mean for young adults not diagnosed with ADHD [28]. This implies that the present sample is symptomatic for ADHD yet has no history of ADHD diagnosis or treatment. No significant differences in demographic variables or baseline ADHD knowledge were found between experimental groups. Full demographic information is presented in Table 1.

**Table 1:** Demographics and descriptives

	Total Sample	Misinformation	Accurate	Control
	*N =* 490	*N =* 155	*N =* 167	*N =* 168
**Year in School**	*N (%)*	*N (%)*	*N (%)*	*N (%)*
1	277 (56.7)	86 (55.5)	98 (58.7)	92 (54.8)
2	119 (24.3)	37 (23.9)	36 (21.6)	46 (27.4)
3	46 (9.4)	13 (8.4)	20 (12.0)	13 (7.7)
4	38 (8.0)	12 (7.8)	12 (7.2)	15 (8.9)
5	10 (2.0)	7 (4.5)	1 (0.6)	2 (1.2)
**Gender**				
Woman/Female	341 (69.6)	105 (67.6)	111 (66.5)	122 (74.0)
Man/Male	147 (30.0)	48 (31.0)	56 (33.5)	43 (25.6)
Other not listed	2 (0.4)	0 (0.0)	0 (0.0)	2 (0.4)
**Race**				
White	292 (59.6)	94 (60.6)	97 (58.1)	101 (60.1)
Asian or Asian American	97 (19.8)	25 (16.1)	36 (21.6)	36 (21.4)
Black or African American	31 (6.3)	11 (7.1)	9 (5.4)	11 (6.5)
Hispanic or Latino/a/x	29 (5.9)	11 (7.1)	9 (5.4)	9 (5.4)
Biracial or Multiracial	26 (5.3)	9 (5.8)	8 (4.8)	9 (5.4)
Middle Eastern/North African or Arab Origin	13 (2.7)	5 (3.2)	6 (3.6)	2 (1.2)
American Indian or Native Alaskan	2 (0.4)	0 (0.0)	2 (1.2)	0 (0.0)
	*M (SD)*	*M (SD)*	*M (SD)*	*M (SD)*
**Age**	19.09 (1.30)	18.98 (1.34)	19.07 (1.12)	19.22 (1.42)
**Social Media Use**	57.36 (11.98)	59.22 (11.51)	57.18 (11.69)	55.86 (12.53)
**ADHD Symptoms**	47.79 (11.61)	48.43 (11.92)	47.44 (12.33)	47.55 (10.63)
**Baseline ADHD Knowledge**	10.00 (1.85)	10.03 (1.52)	9.99 (1.87)	9.99 (2.11)

### Procedure

Participants completed this study in a one-hour in-person experimental research session with measures electronically completed through Qualtrics. The study flow was: completion of baseline ADHD knowledge measure, randomization to content condition, TikTok ADHD-content viewing, completion of remaining survey measures. The primary investigator (also the presenter in all video stimuli) was not present in the room during data collection. The TikTok videos were introduced to participants in the study as follows: “These TikTok videos were recently developed by a new undergraduate student content creator sharing their experiences with ADHD, and we would like to get your opinion about these videos as part of this longer study about ADHD. We have embedded these TikToks directly into the study platform for your convenience.” A research assistant monitored participants and ensured adherence to procedures at each session. The auto-advance and block timer tracking features on Qualtrics and in-person research assistant monitoring were implemented to ensure data quality. Following study completion, participants were debriefed and provided with resources for accurate ADHD information.

## Materials and measures

### Stimuli

TikTok content was developed for this study to provide experimental control (e.g., same presenter, approximate video lengths, topics, format) while manipulating ADHD information disseminated. Use of factitious stimuli in media research is common in media psychology and is particularly useful in determining aspects of media content that may affect user behaviors [29, 30]. Stimuli development is detailed in the Pilot Study sections (see Supplementary File 1). TikTok videos providing ADHD misinformation, accurate ADHD information, and control information were the stimuli for the main study. The presenter in each video was the same young adult who did not claim (or not claim) to have any specific expertise or personal experience with ADHD. Videos in each experimental condition covered a variety of topics (e.g., treatment, symptoms of ADHD, associated features of ADHD) and were developed from a content-analysis of 100 #*ADHD* TikTok videos with high viewership (see OSF or Supplementary Material 1). Control content covered topics related to sleep without mention of ADHD (e.g., sleep hygiene, sleep disorders). Each condition contained multiple videos ranging in duration from 10 s to 90 s, with approximately 12 min total duration of stimuli for each condition.

### Perceived credibility

Participants rated perceived credibility of stimuli on the McCroskey Ethos Measurement Scale, a gold-standard measure to assess perceived expertise and trustworthiness that is applicable to social media content [31]. Items assessing perceived similarity and entertainment were adapted from Munnukka et al. (2016). Perceived expertise (*α =* 0.892), trustworthiness (*α =* 0.854), similarity (*α =* 0.953), and entertainment (*α =* 0.906) subscales were used as predictor variables.

### ADHD knowledge

ADHD knowledge was measured using an adapted version of the Strength of Belief in ADHD Knowledge Scale (SBAKS) and clinical vignettes [26]. The SBAKS contains nine items measuring knowledge and misconceptions of ADHD interventions for children. The wording of SBAKS items was modified to apply to the college student sample (i.e., wording changed from “most children” to “most individuals”). Five items were added to target modern misconceptions of ADHD. Responses were coded dichotomously (1=“*true*”, 2 =“*false*”) for accuracy and on a four-point Likert scale (1=“*just* a guess”, 4=“*I am certain”*) for confidence. Baseline ADHD knowledge was assessed using the SBAKS. The SBAKS was re-administered post-content viewing to calculate direct ADHD knowledge change. The SBAKS demonstrated good internal reliability pre-content-viewing (*α* = 0.848) and post-content-viewing (*α* = 0.896).

Participants also rated whether six clinical vignettes [32] depicted ADHD using the same accuracy and confidence format as the SBAKS. An omnibus post-viewing ADHD knowledge accuracy and omnibus post-viewing ADHD knowledge confidence were created by summing SBAKS and clinical vignettes responses. This omnibus knowledge variable demonstrated excellent internal reliability (*α* = 0.900).

### Treatment-seeking intentions

Treatment-seeking intentions were measured using an adapted version of the Mental Help Seeking Intention Scale (MHSIS), a three-item reliable scale for evaluating mental health help-seeking. The MHSIS items were modified to focus on ADHD treatment-seeking intentions. Six items for evidence-based (e.g., stimulant medication) and non-evidence-based (e.g., dietary changes) treatment-seeking intentions were added. Evidence-based treatment-seeking intentions, non-evidence-based treatment-seeking intentions, and omnibus treatment-seeking intentions (i.e., sum of all nine items; *α* = 0.848) were used as dependent variables in analyses.

### ADHD stigma

ADHD stigma was measured using the ADHD Stigma Questionnaire (ASQ; *α* = 0.927) and was used as a dependent variable in analyses.

### Social media use

Social media use was measured using the Media and Technology Usage and Attitudes Scale (MTUAS), a valid tool to assess media use [33]. Participants also rated hours per week spent on social media platforms using a six-point Likert scale (0 = “*No use*”, 5 = “*More than 5 hours*”). The MTUAS (*α* = 0.850) was used to describe the sample and ensure successful randomization.

### ADHD symptoms

Current ADHD symptoms were measured using the Adult ADHD Self-report Scale (ASRS), which uses a Likert response scale ranged from “0 = never” to “4 = very often” for a total maximum score of 72. The ASRS (*α* = 0.905) was used as inclusion criteria and to describe the sample.

### Demographic information

Age, gender, race, and ethnicity were used descriptively.

### Data analyses

A priori power estimates assuming 95% power to detect significant differences at alpha level 0.05 and medium effect size (*f* = 0.25) across the three experimental group conditions indicated a sample size of 341. Analyses are adequately powered by this study’s 490 participant sample. ANOVA was used to assess between-group differences on baseline ADHD knowledge and descriptive variables to ensure effective randomization. No significant between-group difference in baseline ADHD knowledge (*F*(2,487) = 0.63, *p* =.939, *η*^*2*^ = 0.004) or descriptive variables were found. As such, randomization was successful and baseline ADHD knowledge was not controlled for during analyses.

Multivariate analysis of variance (MANOVA) was used to examine the effect of experimental content condition on all dependent and perceived credibility variables. Repeated measures ANOVAs were used to compare within-group changes in ADHD knowledge accuracy and confidence pre- versus post-content viewing. A series of hierarchical linear regressions were used to examine relationships between experimental conditions, perceived credibility, and dependent variables.

## Results

### Content condition on ADHD knowledge, stigma, and treatment-seeking intentions

#### Between-group comparisons

The MANOVA model for the effect of content condition on ADHD knowledge, stigma, and treatment-seeking intentions was significant (*F*(2,487) = 16.572, *p* <.001, *η*^*2*^ = 0.172, *Pillai’s Trace* = 0.343). Following up with univariate ANOVAs, there was a significant effect of content condition on post-content viewing ADHD knowledge accuracy (*F*(2,487) = 20.383, *p* <.001, *η*^*2*^ = 0.077) and ADHD knowledge confidence (*F*(2,487) = 56.887, *p* <.001, *η*^*2*^ = 0.189). Tukey honestly significant difference post-hoc pairwise comparisons indicated significantly lower post-content viewing ADHD knowledge accuracy in the misinformation group compared to the accurate or control conditions. Participants in the misinformation condition and accurate information condition endorsed greater confidence in ADHD knowledge post-content viewing compared to controls. No significant difference in knowledge accuracy was found between the accurate and control conditions or in knowledge confidence between misinformation and accurate conditions.

There was a significant effect of content condition on evidence-based (*F*(2,487) = 6.773, *p* =.001, *η*^*2*^ = 0.027) and non-evidence-based (*F*(2,487) = 4.041, *p* =.018, *η*^*2*^ = 0.016) treatment-seeking intentions for ADHD. Participants exposed to ADHD misinformation reported higher intentions to seek evidence-based and non-evidence-based treatment compared to controls. There were no significant differences in evidence-based or non-evidence-based treatment-seeking intentions between misinformation and accurate information or accurate information and control conditions.

No significant effects of content condition on ADHD stigma were found (*F*(2,487) = 0.342, *p* =.710, *η*^*2*^ = 0.003). Refer to Table 2 for descriptive statistics of dependent variables by group and Table 3 for ANOVA results.

**Table 2:** Descriptive statistics of ADHD knowledge, ADHD stigma, Treatment-Seeking intentions, and perceived credibility variables by group

	ADHD Misinformation	Accurate Information	Control
	*M (SD)*	*M (SD)*	*M (SD)*
**ADHD Knowledge Variables**			
Pre-Content Viewing SBAKS Accuracy	10.03 (1.52)	9.99 (1.87)	9.99 (2.11)
Post-Content Viewing SBAKS Accuracy	8.71 (1.43)	10.49 (2.40)	9.80 (1.87)
Pre-Content Viewing SBAKS Confidence	34.41 (7.73)	34.58 (8.29)	33.24 (8.49)
Post-Content Viewing SBAKS Confidence	43.81 (10.14)	45.84 (9.10)	35.19 (9.80)
Post-Content Viewing ADHD Knowledge Accuracy	13.17 (1.84)	14.53 (2.02)	14.29 (2.20)
Post-Content Viewing ADHD Knowledge Confidence	43.81 (10.14)	45.84 (9.01)	35.19 (9.80)
**ADHD Stigma Variable**			
ADHD Stigma	60.06 (12.60)	60.57 (12.91)	61.18 (11.91)
**ADHD Treatment-Seeking Intentions Variables**			
Treatment-Seeking Intentions	33.74 (7.66)	33.52 (8.22)	32.80 (6.52)
Evidence-Based Treatment-seeking	8.94 (2.47)	8.26 (2.64)	7.94 (2.32)
Non-Evidence-Based Treatment-seeking	9.72 (2.55)	9.28 (2.72)	8.92 (2.48)
**Perceived Credibility Variables**			
Total Perceived Credibility	89.07 (24.38)	88.70 (22.54)	97.13 (24.22)
Perceived Expertise	17.04 (6.58)	15.18 (6.07)	16.61 (6.11)
Perceived Trustworthiness	36.45 (8.78)	34.08 (9.62)	38.94 (9.61)
Perceived Entertainment	20.15 (6.74)	17.45 (8.45)	22.99 (7.49)
Perceived Similarity	18.23 (7.79)	20.29 (6.29)	18.29 (6.90)

Note: SBAKS, Strength of Belief in ADHD Knowledge Scale, ADHD Knowledge Accuracy and ADHD Knowledge Confidence are omnibus variables created from accuracy/confidence in the SBAKS and ADHD vignette items; Treatment-Seeking Intentions is an omnibus variable that contains general ADHD treatment-seeking intentions, along with evidence-based and non-evidence-based treatment-seeking intentions; Total maximum scores for each perceived credibility subscale differ; total maximum scores for expertise is 35, for trustworthiness is 84, and for enterinament and similarity is 35

**Table 3:** Pairwise comparisons of content condition on ADHD knowledge, stigma, Treatment-Seeking intentions, and perceived credibility

	Overall Model				Pairwise Comparisons									
					Misinformation vs. Accurate			Misinformation vs. Control			Accurate vs. Control			
	*F*	*p*	*η* ^*2*^		*Mdiff*	*p*	*95% CI*	*Mdiff*	*p*	*95% CI*	*Mdiff*		*p*	*95% CI*
Post- Knowledge Accuracy	20.383	> 0.001	0.077		-1.37	< 0.001	-1.90, -0.83	-1.12	< 0.001	-1.65, − 0.586	0.25		0.506	-0.27, 0.769
Post- Knowledge Confidence	56.887	> 0.001	0.189		-2.04	0.143	-4.58, 0.50	8.62	< 0.001	6.08, 11.15	10.65		< 0.001	8.17, 13.14
ADHD Stigma	0.342	0.710	0.001		-1.35	0.564	-4.72, 1.49	-1.62	0.440	-4.73, 1.49	-0.27		0.976	-3.32, 2.77
Treatment-Seeking Intentions	0.708	0.493	0.003		-0.22	0.962	-2.19, 1.75	0.80	0.607	-1.17, 2.76	1.02	0.869		-0.68, 1.06
EB Treatment Intentions	6.773	0.001	0.027		0.79	0.093	-0.10, 1.68	0.98	0.027	0.09, 1.86	0.19		0.869	-0.68, 1.06
Non-EB Treatment	4.041	0.018	0.016		-0.38	0.392	-1.05, 0.30	0.81	0.011	-1.48, -0.15	0.44		0.281	-0.24, 1.11
**Perceived Credibility**														
Total Perceived Credibility	5.889	0.003	0.024		-0.63	0.969	-6.85, 5.59	-8.05	0.007	-14.3, -1.84	-7.42		0.012	-13.5, -1.33
Perceived Expertise	3.926	0.020	0.016		1.86	0.021	0.22, 3.50	0.43	0.809	-1.20, 2.07	-1.43		0.093	-3.03, -0.18
Perceived Trustworthiness	11.291	> 0.001	0.044		2.27	0.076	-0.18, 4.73	-2.59	0.036	-5.04, -0.14	-4.86		< 0.001	-7.26, -2.45
Perceived Entertainment	21.655	> 0.001	0.082		5.54	< 0.001	-7.52, -3.56	-2.70	0.004	-4.69, -0.72	-2.84		0.002	-4.78, -0.89
Perceived Similarity	4.027	0.018	0.016		-2.05	0.024	-3.89, -0.22	-0.36	0.891	-2.19, 1.48	-1.70		0.069	-3.50, 0.10

Note: CI, Confidence Interval; EB, Evidence-Based; Mdiff, Mean Difference; Post-, Post-Content Viewing; Knowledge and Accuracy represent the total ADHD Knowledge and Accuracy scores

#### Within-group comparisons

Participants exposed to accurate ADHD-content exhibited significantly increased ADHD knowledge accuracy (*F*(1,166) = 6.620, *p* =.011, *η*^*2*^ = 0.039) and confidence in their ADHD knowledge (*F*(1,166) = 357.055, *p* <.001, *η*^*2*^ = 0.701) pre- to post-content viewing.

Participants exposed to ADHD misinformation exhibited significantly less accurate (*F*(1,154) = 89.819, *p* <.001, *η*^*2*^ = 0.368) ADHD knowledge and reported significantly increased confidence in their knowledge (*F*(1,154) = 206.134, *p* <.001, *η*^*2*^ = 0.590) post-content viewing versus baseline.

**Fig. 1:**
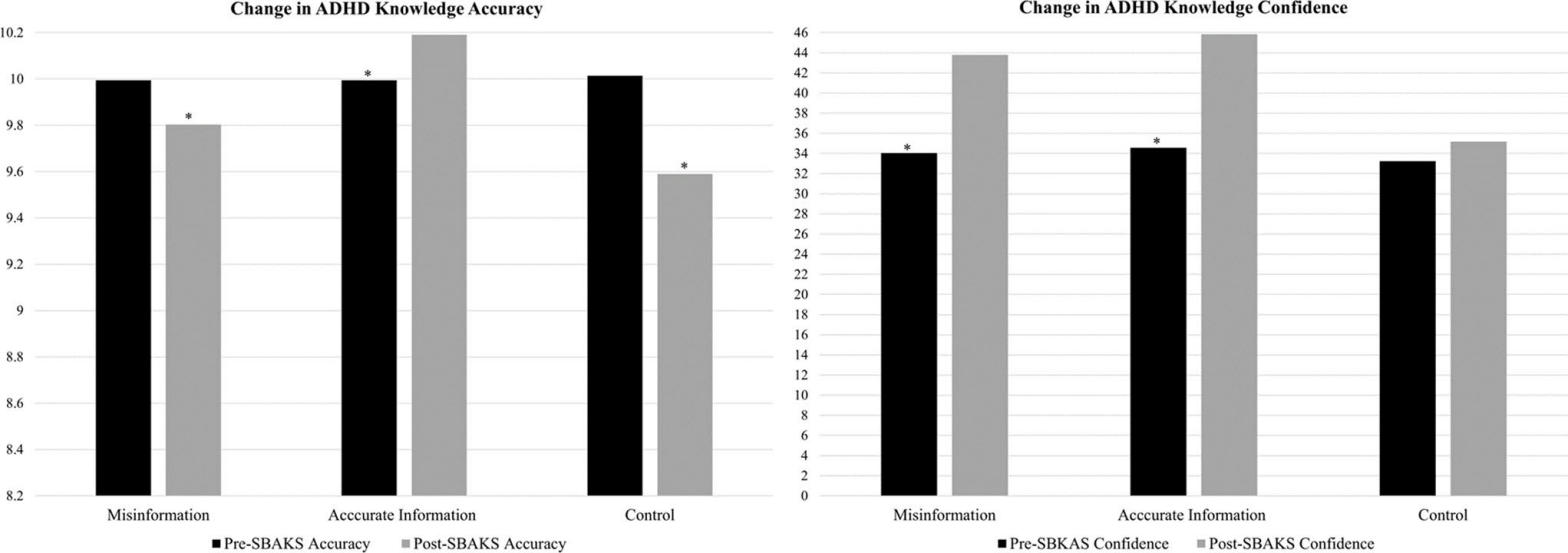
Mean pre- and post-content viewing ADHD knowledge accuracy and confidence by group. *Note*: SBAKS, Strength of Beliefs in ADHD Knowledge Scale; ADHD knowledge accuracay depicted in this figure only denotes knowledge measured via SBAKS

Participants in the control condition possessed significantly less accurate ADHD knowledge (*F*(1,167) = 17.060, *p* <.001, *η*^*2*^ = 0.096) but reported no significant change in their knowledge confidence (*F*(1,167) = 1.533, *p* =.214, *η*^*2*^ = 0.009) post-content viewing versus baseline. Figure 1 depicts the mean changes in SBAKS pre- and post-content viewing by condition.

### Differences in perceived credibility

The MANOVA model for content condition on perceived credibility variables was significant (*F*(2,487) = 14.051, *p* <.001, *η*^*2*^ = 0.104, *Pillai’s Trace* = 0.208). Content condition was significantly related to total perceived credibility (*F*(2,487) = 5.889, *p* =.003, *η*^*2*^ *=* 0.024) and each perceived credibility subscale. Table 2 provides descriptive statistics of perceived credibility by group. Table 3 provides ANOVA results.

There was a significant effect on content condition on perceived expertise (*F*(2,487) = 3.926, *p* =.020, *η*^*2*^ *=* 0.016). Participants exposed to misinformation rated content as significantly more expert compared to participants exposed to accurate information. No significant differences in perceived expertise between misinformation and control conditions or accurate and control conditions were found.

There was a significant effect of content condition on perceived trustworthiness (*F*(2,481) = 11.291, *p* <.001, *η*^*2*^ *=* 0.044). Participants in the misinformation and accurate conditions rated the content to be significantly less trustworthy compared to controls. There was not a significant difference in perceived trustworthiness between the misinformation and control conditions or accurate and control conditions.

There was a significant effect of content condition on perceived entertainment (*F*(2,487) = 21.655, *p* <.001, *η*^*2*^ *=* 0.082). Participants exposed to misinformation rated the content as significantly more entertaining than participants exposed to accurate information and significantly less entertaining than control participants. Participants in the control condition rated the content as significantly more entertaining compared to the accurate condition.

There was a significant effect of content condition on perceived similarity (*F*(2,487) = 4.027, *p* =.018, *η*^*2*^ *=* 0.016). Participants exposed to misinformation rated the content significantly lower in similarity compared to the accurate condition. No significant differences in perceived similarity between misinformation and control conditions or accurate and control conditions were found.

### Perceived credibility on ADHD knowledge, stigma, and treatment-seeking intentions

The regression model for post-ADHD knowledge accuracy was significant at step 1 (*F*(2,487) = 14.966, *p* <.001, *R*^*2*^ = 0.002) and step 2 (*F*(4,484) = 6.106, *p <*.001, *R*^*2*^ = 0.002). Perceived entertainment and perceived similarity were significantly associated with post-content-viewing ADHD knowledge accuracy. No other credibility variables were significantly associated with post-ADHD knowledge accuracy.

The regression model for ADHD stigma was not significant at step 1 (*F*(2,487) = 0.673, *p* =.412, *R*^*2*^ = 0.001) and was significant at step 2 (*F*(4,484) = 2.667, *p* =.022, *R*^*2*^ = 0.001). Perceived entertainment was negatively associated with ADHD stigma.

Regression models for overall ADHD treatment-seeking intentions (*F*(2,487) = 1.024, *p* =.312, *R*^*2*^ = 0.002) and non-evidenced based treatment-seeking intentions (*F*(2,487) = 3.098, *p* =.079, *R*^*2*^ = 0.006) were both non-significant at step (1) The regression model for evidence-based treatment-seeking was significant at step 1 (*F*(2,487) = 5.477,*p* =.020, *R*^*2*^ = 0.008). Regression models for overall (*F*(4,484) = 9.567, *p* <.001, *R*^*2*^ = 0.090), evidence-based (*F*(4,484) = 9.399, *p* <.001, *R*^*2*^ = 0.089), and non-evidenced-based treatment-seeking intentions (*F*(4,484) = 5.935, *p* <.001, *R*^*2*^ = 0.058) were significant at step (2) Perceived entertainment was negatively associated with overall, evidence-based, and non-evidence-based treatment-seeking intentions. Perceived trustworthiness was negatively associated with overall treatment-seeking intentions. See Table 4 for full hierarchical linear regression results.

**Table 4:** Hierarchical linear regression of content condition and perceived credibility on ADHD knowledge, stigma, and Treatment-Seeking intentions

	*R* ^2^	ΔR^2^	B	β	t	*p*
**Post-Content Viewing ADHD Knowledge Accuracy**						
Step 1	0.030	0.030				
Content Condition			− 0.292	− 0.172	-3.869	> 0.001
Step 2	0.059	0.030				
Perceived Expertise			− 0.031	− 0.092	-1.596	0.113
Perceived Trustworthiness			− 0.002	− 0.009	− 0.155	0.877
Perceived Entertainment			0.035	0.129	2.217	0.027
Perceived Similarity			0.033	0.110	2.126	0.034
**ADHD Stigma**						
Step 1	0.001	0.001				
Content Condition			− 0.362	− 0.038	− 0.820	0.412
Step 2	0.028	0.027				
Perceived Expertise			− 0.218	− 0.115	-1.891	0.059
Perceived Trustworthiness			0.090	0.073	1.181	0.238
Perceived Entertainment			− 0.202	− 0.134	-2.219	0.027
Perceived Similarity			0.040	0.023	0.436	0.663
**Treatment-Seeking Intentions**						
Step 1	0.002	0.002				
Content Condition			0.275	0.046	1.012	0.312
Step 2	0.090	0.088				
Perceived Expertise			− 0.025	− 0.021	− 0.317	0.711
Perceived Trustworthiness			− 0.108	− 0.137	-2.370	0.018
Perceived Entertainment			− 0.204	− 0.215	-3.740	> 0.001
Perceived Similarity			0.032	0.030	0.592	0.554
**Evidence-Based Treatment-seeking Intentions**						
Step 1	0.008	0.008				
Content Condition			0.245	0.090	1.984	0.048
Step 2	0.089	0.081				
Perceived Expertise			0.017	0.032	0.556	0.579
Perceived Trustworthiness			− 0.038	− 0.108	-1.860	0.063
Perceived Entertainment			− 0.111	− 0.257	-4.470	> 0.001
Perceived Similarity			0.012	0.024	0.478	0.633
**Non-Evidence-Based Treatment-seeking Intentions**						
Step 1	0.006	0.006				
Content Condition			0.166	0.080	1.760	0.079
Step 2	0.058	0.052				
Perceived Expertise			− 0.033	− 0.080	-1.380	0.168
Perceived Trustworthiness			− 0.021	− 0.077	-1.313	0.190
Perceived Entertainment			− 0.044	− 0.133	-2.273	0.023
Perceived Similarity			0.007	0.018	0.350	0.726

## Discussion

This is the first study to use an experimental design to investigate the impacts of TikTok ADHD misinformation on ADHD knowledge, stigma, and treatment-seeking intentions in college students using theoretical frameworks. Exposure to ADHD misinformation decreased accurate ADHD knowledge and increased confidence in ADHD knowledge. Compared to exposure to accurate information, misinformation was associated with lower overall ADHD knowledge. Compared to the control condition, exposure to misinformation was associated with lower overall ADHD knowledge, greater confidence in ADHD knowledge, and higher treatment-seeking intentions. The finding of low knowledge and increased treatment-seeking intentions is inconsistent with IMB model [11]. Nonetheless, these results demonstrate the potential effects of ADHD misinformation on ADHD knowledge and treatment-seeking-intentions in emerging adults.

### Impact of misinformation on knowledge, confidence, and treatment-seeking intentions

Despite possessing lower ADHD knowledge, participants in the misinformation condition were as confident in their ADHD knowledge as participants in the accurate condition. This combination of lower knowledge and higher confidence is consistent with the Dunning-Kruger effect, a common cognitive bias that indicates that individuals with lower knowledge often overestimate their knowledge [34]. Although no research has considered the Dunning-Kruger effect in ADHD treatment-seeking intentions, the effect is well-documented in healthcare decision-making research [35]. In general, individuals with low health knowledge rate equal or greater confidence in their health knowledge yet are more likely to engage in poor health behaviors compared to those with high health knowledge [35]. College students with high confidence in their knowledge of health information are more likely to self-diagnose a health condition and seek out medication usage without physician guidance [36]. The Dunning-Kruger effect illustrates that individuals with low knowledge and high confidence are likely to reduce their reliance on professionals and place higher emphasis on information from non-experts [36, 37]. This effect may explain why exposure to misinformation and subsequent low ADHD knowledge/high confidence in that knowledge is associated with higher treatment-seeking intentions, which is antithetical to the predictions from the IMB model.

On an individual level, low ADHD knowledge and high confidence in knowledge may lead to unhelpful health decisions. Individuals exposed to misinformation reported higher intentions to seek non-evidence-based treatments compared to control participants. Engagement in non-evidence based treatments is harmful due to wasted resources (e.g., time, money) and false hope [38], yet individuals exposed to misinformation online may view non-evidence based treatments and perceive them as viable options to manage ADHD symptoms. For individuals with clinical levels of ADHD symptoms, engagement in non-evidence-based treatments may lead to self-diagnosis (e.g., “*I must not have ADHD if bilateral stimulation treatment is not helping*”) and ultimately deter at-risk individuals from seeking evidence-based treatments [36]. This “self-diagnosis” or self-identified ADHD may be developed and subsequently reinforced by exposure to ADHD information (and misinformation) online [39]. An example of this “self-diagnosis” cycle may be that an individual views information/misinformation about ADHD online, relates that information to their own experience, then begins to pay greater attention to their behaviors that align with the information online [39]. Exposure to misinformation is likely to prime individuals to “symptoms” they may experience, thus framing their behaviors in the context of a diagnosis/health condition and overreporting symptoms [40]. Through repeated exposure to ADHD content online, individuals may be more apt to relate their behaviors to a diagnostic label of ADHD [39]. This self-diagnosis of ADHD may impede warranted health-seeking by reduced reliance on healthcare professionals [36, 37] and/or promote unwarranted treatment-seeking.

Seeking evidence-based treatment for ADHD based on misinformation may further overburden a taxed ADHD healthcare system [13] and contribute to unnecessary treatment-seeking among individuals without clinical ADHD. The present results suggest that, in part, exposure to misinformation may influence individuals to seek evidence-based treatment predicated on a “self-diagnosis” of ADHD. Given the scarcity of stimulant medication and low access to ADHD assessment in the United Kingdom and United States [13, 14], superfluous treatment-seeking constitutes a public health concern. The volume of individuals seeking these services may be increased by exposure to misinformation, thus creating barriers for individuals with warranted ADHD concerns, based upon accurate knowledge, to obtain treatment.

### Role of perceived entertainment

Higher perceived entertainment was related to increased ADHD knowledge, lower ADHD stigma, and lower treatment-seeking intentions. This finding suggests that perceived entertainment is the most pertinent factor of perceived credibility for altering ADHD knowledge and treatment-intentions. Previous research demonstrates that entertainment is germane to consumer behaviors. Perceived entertainment is associated with increased cognitive engagement [41], and viewers may be more likely to remember, believe, or act on information from entertaining content [42]. Entertaining content tends to be more popular [43], and entertainment is a top motivator for sharing misinformation [19]. Although social media often presents inaccurate ADHD information [6], its entertainment value can lead to widespread sharing and repeated exposure to misinformation [43], which may consequently decrease public ADHD knowledge.

### Clinical and practical implications

Because perceived entertainment was significantly associated with ADHD knowledge, increasing the entertainment value of high-quality, accurate ADHD content is an evident step to improving public knowledge regarding ADHD.Beyond improving access to accurate ADHD information, combatting the spread of and belief in ADHD misinformation is vital. Debunking, pre-bunking, and nudging are evidence-based methods to combat the spread of online misinformation [44]. Debunking involves post-hoc corrections of misinformation, using clear, detailed explanation refuting inaccurate information [45]. Pre-bunking prevents initial belief in misinformation by explicitly warning individuals about common misinformation and pre-emptively refuting it [46]. Nudging (e.g., providing brief pop-up/disclaimer messages) has also demonstrated efficacy in dissuading individuals from sharing or believing in misinformation online [47]. Simultaneously engaging in debunking, pre-bunking, and nudging against ADHD misinformation while increasing availability of accurate, entertaining ADHD information online may help to improve mental health literacy regarding ADHD. However, a recent study discerned that debunking was ineffective against individuals who “self-diagnosed” ADHD, potentially indicating a need for proactive measures to emphasize pre-bunking [48]. National organizations and other large-scale institutions likely have the greatest resources to disseminate large-scale media campaigns against ADHD misinformation. On smaller scales, mental health professionals who work with patients seeking ADHD treatment or assessment should familiarize themselves with common ADHD misinformation by engaging with this online content. Mental healthcare providers who are familiar with common ADHD misconceptions may be better equipped to effectively pre-bunk and debunk misinformation that patients may encounter online [45].

Social media “influencers” have large followings, and their messages often influence decision-making (e.g., health decisions, spending decisions) amongst their followers [49]. As such, the unintentional spread of misinformation by social media influencers, likely motivated by a desire to entertain their audiences and grow their following [19], may inadvertently sway their followers into engaging in ineffective or unnecessary treatment-seeking. Policies by social media platforms could require content creators to engage in trainings that inform creators about the impacts of content messaging on viewers, and targeted trainings on health literacy might be considered for content creators who are identified as creating content on health-related topics (e.g., ADHD). Importantly, a recent field intervention study that provided TikTok influencers with training on how to effectively prompt evidence-based mental health communication yielded preliminary evidence that content creator focused interventions may be a feasible way to reduce the spread of misinformation and increase access to accurate mental health information online [50]. Moreover, social media platforms could implement nudging to prior to an individual posting content containing misinformation. Algorithms and artificial intelligence provides the ability for social media posts to be screened and evaluated for misinformation [51], and a nudge altering the content creator prior to posting (e.g., “*This post is estimated to contain over 80% misinformation. Consider not posting*.”) could be an additional measure against the spread of misinformation [47, 52].

### Limitations and future directions

Videos developed for this study highly resembled the format and topics native to TikTok, and the study design aimed to maximize experimental control while retaining ecological validity. Nonetheless, future research could study in situ viewing of TikTok videos to investigate how native TikTok content influences ADHD knowledge and treatment-seeking intentions. Although this study found significant effects of content condition and perceived entertainment, the overall regression models explained a small percentage of variability. One explanation is that participants were exposed to one 12-minute session of ADHD TikTok videos. In real-world scenarios, individuals are more likely encounter ADHD TikTok videos in a more spontaneous and sporadic rate over the course of various TikTok sessions. Repeated, chronic exposure to messaging is more likely to engender changes in the viewer [41] and may increase predictive model strength. It is possible that inundation of ADHD information from TikTok, due to algorithms pushing content that viewers have previously engaged with, creates greater impacts on viewers’ ADHD knowledge and treatment-seeking intentions beyond what was demonstrated in one study session.

Because no between group differences in ADHD symptoms were found and ADHD symptoms were not in a priori hypotheses, effects of ADHD symptoms on dependent variables were not investigated. However, individuals with higher levels of ADHD symptoms may have higher baseline exposure to ADHD (mis)information by proactively seeking content to understand their symptoms prior to study completion. Individuals who have personal experiences with individuals diagnosed with ADHD may possess different baseline ADHD knowledge and stigma compared to those without personal experiences with ADHD [53]. Further, there was no significant effect of content condition on ADHD stigma. Research as to the effects of social media on mental health stigma, broadly, have exhibited mixed findings [21, 54], such that social media both perpetuates misconceptions and stigma surrounding mental health [21, 54] and provides increased awareness and opportunities to gain peer support online [55]. Reducing stigma and disseminating information are not mutually exclusive outcomes; it is plausible that as one becomes more accepting/aware of ADHD, they might also be more prone to engaging ADHD content, potentially exposing them to misinformation, in an effort to become more informed. Therefore, the relationship between ADHD TikTok content and ADHD stigma is likely bidirectional and complex, and future research may seek to more thoroughly investigate how social media impacts ADHD stigma among college students. Likewise, investigating the role of ADHD symptoms and previous experience with ADHD on outcome may help further explain additional variability in the model.

This present study’s experimental design demonstrated that exposure to ADHD misinformation decreases ADHD knowledge, increases confidence in this knowledge and increased intentions to seek evidence-based and non-evidence-based treatments for ADHD. Low ADHD health literacy may lead to frivolous ADHD treatment-seeking predicated on misconceptions, consequently exacerbating the public health crisis of the ADHD assessment and treatment shortage. Findings from this study provide an essential first-step in understanding the harms of TikTok ADHD misinformation on individual and public levels.

## Electronic supplementary material

Below is the link to the electronic supplementary material.

Supplementary Material 1

## Data Availability

Raw data from this study can be made available by contacting the corresponding author. All procedures and stimuli for this study are available on OSF (https://osf.io/v5kwr/?view_only=c3090e2e6fd14378ba7459c9380a51dd).
