# Supplementary material for: Misinformation mayhem: the effects of TikTok content on ADHD knowledge, stigma, and treatment-seeking intentions
Source: Eur Child Adolesc Psychiatry. 2025 Jun 5;34(11):3521–33. doi: 10.1007/s00787-025-02769-8 (PMC12647241; doi:10.1007/s00787-025-02769-8)
Supplement: Supplementary file 1 — Supplementary Material 1 [file 787_2025_2769_MOESM1_ESM.docx]

## Supplementary Material 1

## **Pilot Study**

The pilot study involved stimuli development and pilot testing of stimuli and study measures. The goal of stimuli development was to systematically develop misinformation and accurate ADHD content that highly resembled the video content and style present in the TikTok ecosystem. The misinformation content aimed to include the common themes and inaccuracies found on TikTok whereas the accurate information aimed to provide evidence-based information. Pilot testing was then completed to assess: 1) that stimuli from all conditions were deemed to be similarly realistic to participants; 2) if ADHD knowledge changes following content-viewing; 3) if the internal reliabilities for self-report measures that were altered from their original form (described in detail in the Main Study Measures) were acceptable.

### ***Stimuli Development***

Content mimicking the format and content of popular TikTok ADHD videos was developed. Scripts developed for misinformation and accurate information conditions covered topics such as ADHD symptoms and treatment. Scripts developed for the control condition covered information about a non-ADHD related health topic (i.e., sleep). Scripts intended for the misinformation content conditions were based on common ADHD misinformation identified via a systematic content analysis of popular TikTok videos with the “ADHD” tag. The systematic content analysis constituted viewing 100 ADHD TikTok videos with over one million views each, rating each video for content accuracy, and scribing the scripts, scene direction, and music used in each video.

The procedure for assessing video accuracy followed the same methods described in a previous content analysis of ADHD TikTok videos.^5^ The most prevalent misinformation topics among these videos were identified through reviewing video scripts. The topics, information, and scripts collected from this content analysis were adapted to create the misinformation condition scripts. Scripts intended for the accurate condition were developed based on empirical research articles and *DSM-5-TR* criteria for ADHD due to insufficient accurate information on TikTok, per content analysis results. Scripts intended for the control condition were developed based on *DSM-5-TR* criteria for sleep disorders and empirical research regarding sleep. The scene directions, video graphics, and music used in actual TikTok videos were heavily incorporated into the videos for each condition to create content that highly resembles the content organic to TikTok.

Content scripts were assessed for quality by two advanced clinical psychology doctoral students and a licensed clinical psychologist with expertise in ADHD using the Patient Education Materials Assessment Tool (PEMAT) for Printable Materials and the *Journal of American Medical Association* benchmark criteria for content validity. Scripts for the accurate conditions were rated as statistically more accurate (*M*=3.74, *SD*=0.64; *p<*.001) compared to scripts for the misinformation conditions (*M*=1.47, *SD*=0.63) on the following scale: “*1 – entirely irrelevant and inaccurate*” to “*4 – very relevant and accurate*”. Inter-rater reliability was substantial for accurate scripts (*κ*=.699) and moderate for misinformation scripts (*κ=*.470). This level of inter-rater reliability is considered to be acceptable for stimuli development.^29^ Each evaluator provided script feedback and revisions, which were integrated prior to video recordings.

Videos for misinformation, accurate information, and control scripts were performed by the same young adult. The young adult introduced herself as an undergraduate student and dressed in casual attire, mimicking the primary demographic of content creators (i.e., young adults) on TikTok. Multiple videos ranging in duration from 10 seconds to 90 seconds were recorded for each condition. The total duration of stimuli for each condition was approximately 12 minutes, which was selected as it is the average TikTok session length.^30^ Content was then pilot tested.

### ***Pilot Study Participants, Procedures, and Measures***

A total of 21 college students from BLINDED FOR REVIEW were recruited to complete a pilot testing. The majority of pilot study participants were in their first year of college (45%) and identified as White (80%) and female (80%). Their mean age was 18.81 (*SD=*.95). Participants were randomly assigned to one of three content conditions (i.e., accurate information, misinformation, control). Seven participants were in each condition, and no significant differences in demographic variables were noted between groups.

Participants followed the same procedure as the main study. Briefly, participants completed a baseline measure of ADHD knowledge, viewed TikTok content per random assignment to study condition, then completed post-content viewing survey measures. Two items were used to determine if there were significant differences in perceptions of the videos between groups. These items asked participants to rate the accuracy of information presented on a 7-point Likert scale (1 = “*not at all accurate”* to 7 = “*extremely accurate*”) and if they believed the videos presented were real TikTok videos on a 7-point Likert scale (1 = “*definitely not real”* to 7 = “*definitely real*”). All measures used in the pilot study are fully detailed in the Main Study Measures section below.

### ***Pilot Study Results***

There were no significant between-group differences in perceptions of the videos’ realness (*F*(2,19)=.441, *p*=.651) or accuracy (*F*(2,19)=.633, *p*=.543). Repeated measures analysis of variance (ANOVA) was used to determine if ADHD knowledge changed within-subjects after viewing content (i.e., pre-content viewing ADHD knowledge compared to post-content viewing ADHD knowledge within-subjects). This analysis was used to determine if the brief exposure to TikTok content engendered change in ADHD knowledge, thus demonstrating feasibility for the main study. Significant changes in ADHD knowledge pre- compared to post-content viewing were found among the whole sample (*F*(1,20)=16.855, *p*=.001). Results provided preliminary evidence that the experimental manipulation of content condition impacted ADHD knowledge and that participants did not significantly differ in their beliefs that these videos were real or accurate by condition. Acceptable internal reliability was noted in all survey measures. Therefore, the main study proceeded without further refinements of stimuli or measures.
